# Editorial: Application of nano/biotechnology in the detection of food safety and spoilage

**DOI:** 10.3389/fnut.2023.1154898

**Published:** 2023-02-28

**Authors:** Tarun Belwal, Yizhong Shen, Seid Jafari, Xingyu Lin

**Affiliations:** ^1^State Key Laboratory of Fluid Power and Mechatronic Systems, College of Biosystems Engineering and Food Science, Fuli Institute of Food Science, Zhejiang University, Hangzhou, China; ^2^Key Laboratory for Agricultural Products Processing of Anhui Province, School of Food and Biological Engineering, Hefei University of Technology, Hefei, China; ^3^Faculty of Food Science and Technology, Gorgan University of Agricultural Sciences and Natural Resources, Gorgan, Iran; ^4^Key Laboratory of Agro-Products Postharvest Handling of Ministry of Agriculture and Rural Affairs, Zhejiang University, Hangzhou, China

**Keywords:** nano/biotechnology, food safety, electrochemical analysis, optical analysis, signal amplification

Nanotechnology is an emerging technology that utilizes the characteristics of atomic and molecular structures and their interaction principles at the nanoscale and microscale. A lot of nanotechnology research is focused on the field of biological science, while the application of nanotechnology and biotechnology in the field of food science mainly focuses on food safety and spoilage, which can greatly improve the speed, efficiency, and accuracy of food safety and spoilage detection, simplify operation steps, and save manpower and material resources.

This Research Topic provides the latest trends on the different analytical methods applying nano/biotechnology for the detection of hazardous substances in food: electrochemical-based food analysis methods and optical-based food analysis methods.

Electrochemical sensors have high sensitivity, accuracy, and stability for the monitoring of food safety and spoilage and have been widely used in the field of food science. Vertically-ordered mesoporous silica films (VMSF) are attractive nanoporous materials that have ultra-small, ordered, and uniform nanopores perpendicular to the electrode substrate, showing excellent molecular selectivity and anti-fouling ability, which can be directly applied in complex samples. Guanine, which is abundant in foods and drinks, such as seafood and beer, eventually metabolizes into uric acid in the body, possibly causing gout. Yang L. et al. developed a sensitive electrochemical method for the detection of guanine by integrating VMSF/ITO and tris(2,2'-bipyridine) ruthenium (III) [${\text{Ru(bpy)}}_{3}^{2+}$] redox media. The electrostatic accumulation of ${\text{Ru(bpy)}}_{3}^{2+}$ by VMSF can act as an electron shuttle between the surface of guanine and the underlying ITO. Thus, it can be used to quantify guanine. In addition to the direct electrochemical (EC) method, electrochemical luminescence (ECL) technology also shows outstanding application prospects in the field of food analysis. Luo et al. first established an EC and ECL two-mode detection method for antimicrobial peptide analysis by combining the specific recognition from antibacterial peptides and the signal amplification effect from VMSF. ${\text{Ru(bpy)}}_{3}^{2+}$@ liposomes are used as signal probes, which can be enriched and sensitively detected by VMSF/ITO electrodes when the ${\text{Ru(bpy)}}_{3}^{2+}$ leaks from nisin-damaged liposomes. The nanoporous material-modified electrode in the confined space can detect the Faraday current to sense the analyte, while the single nanopore can also detect the analyte signal due to ionic current changes. In another study, Yang T. et al. prepared a low aspect-ratio SiN pore with PDMS coating that has high temporal-spatial resolution and can be used to observe the bacterial translocation “event”. According to the changes in the electric pulse signals generated by different bacteria passing the pore, three common foodborne pathogens can be identified, such as *Salmonella enterica, Listeria monocytogenes*, and *Escherichia coli*.

Optical analysis, which is based on the interaction between electromagnetic radiation and matter, plays an important role in food analysis. Nanomaterials can significantly improve the signal strength of food-analyzed substances. Wang et al. developed a new method for the determination of trace Ag(I) by spectrophotometry and Rayleigh scattering (RRS). Ag(I) can react with erythrosine to form nanoparticles in pH 4.4–4.6, leading to a lower absorbance and higher RRS signal, thus the quantitative determination of Ag(I) content was realized. Lv et al. used AuMOF as a nanoprobe to establish a new RS-ET method for detecting sulfur dioxide concentration in food. When basic rhodopsin (BF) is on the surface of AuMOF, RRS intensity at 330 nm decreases, and sulfite can react with BF to form a colorless product (SBF), resulting in an enhanced RRS peak. Therefore, it can be used for quantitative analysis of sulfur dioxide. Zhi et al. established a novel MXene catalytic fluorescence/absorption two-mode aptamer sensor to detect traces of Pb^2+^. Ti_3_C_2_ nanosheet (NS) can catalyze the oxidation of TMB, which can lead to an intense fluorescence peak at 415 nm and an absorption peak at 295 nm, but the Pb^2+^ aptamer (Apt_pb_) that adsorbed on the surface of NS can inhibit its catalytic activity. When target Pb^2+^ is added, it specifically binds with Apt_pb_, leading to the release of Apt_pb_ from NSs, and the signal is enhanced. Shahdeo et al. developed a microfluidic colorimetric detection device coupled with AuNPs and aptamers to detect Ochratoxin A (OTA). AuNPs were combined with an OTA-specific 36-mer aptamer, and the size of AuNPs changed in the presence of an OTA analyte. The absorbance ratios of A_630_ and A_520_ were determined to quantitate OTA.

In summary, the studies in this collection showcase the latest advances in the application of nanomaterials to enhance the signal of electrochemical and optical analysis. The research has demonstrated that nano/biotechnology can significantly improve the analytical performance of food analytical methods. We believe that nano/biotechnology has great potential in the development of food safety and spoilage detection technology and these articles may provide inspiration for future research.

## Author contributions

TB, YS, SJ, and XL are responsible for the management of the whole issue. TB wrote the introduction and the conclusion. XL wrote the central part with comments to the cited papers. YS and SJ revised the paper. All authors listed have made a substantial, direct, and intellectual contribution to the work and approved it for publication.

